# Psychometric properties of the Malay version Women’s Views of Birth Postnatal Satisfaction Questionnaire using the Rasch measurement model: a cross-sectional study

**DOI:** 10.1186/s12884-021-04184-8

**Published:** 2021-10-22

**Authors:** Mohd Noor Norhayati, Adnan Fatin Imtithal, Yunus Nor Akma

**Affiliations:** Department of Family Medicine, School of Medical Sciences, Health Campus, Universiti Sains Malaysia, 16150 Kubang Kerian, Kelantan Malaysia

**Keywords:** Postnatal, Postpartum, Psychometric, Rasch model, Satisfaction

## Abstract

**Background:**

Maternal satisfaction must be assessed in order to reflect the quality of care, which is considered an outcome of healthcare services. It can also be used to contrast and compare satisfaction with various care models or service configuration or to assess overtime changes. The purpose of this study was to investigate the psychometric properties of the Malay version Women’s Views of Birth Postnatal Satisfaction Questionnaire (WOMBPNSQ) based on the Rasch scale model of labour satisfaction.

**Methods:**

This is a cross-sectional study. Postpartum women were identified from a tertiary hospital and evaluated at 1-month postnatal period using WOMBLSQ. The Rasch model was used to investigate the reliability, unidimensionality, item and person misfits and distribution map.

**Results:**

A total of 195 women were involved. The Rasch analysis revealed that the 30 items had a high level of reliability at 0.99 and item separation at 9.02. It has a low level of reliability at 0.45 and persons separation at 0.90. All the items are considered fit. Five people have most misfitting response strings based on item IPS_Q15, ‘*I was given little advice on contraception following the birth of my baby’*, but extremely trivial differences were found in the parameter estimates after refitting the model. The more difficult item to endorse satisfaction is item CA_Q17 *‘I was given little advice on contraception following the birth of my baby’*.

**Conclusions:**

The WOMBLSQ tested in postpartum women proved to have high item reliability index but with an adequate sample. The analysis shows that the 30 items target the right form of respondents, have similar latent characteristics of postpartum women and a shared sense of satisfaction. For future improvement, more difficult items endorsing satisfaction should be created, and the common items in which satisfaction is expected should be reduced.

**Supplementary Information:**

The online version contains supplementary material available at 10.1186/s12884-021-04184-8.

## Background

Understanding maternal satisfaction is important as it forms a proxy measure for quality of care. There is a lack of elements to define the quality treatment comprehensively, and research has shown that increasing the coverage of critical services is inadequate to minimize maternal mortality and serious morbidity. There is a complex interplay between treatment experience and the outcome of pregnancy [1]. As an alternative to reflecting the quality of care, patient-centred measures such as maternal satisfaction was considered and is regarded as a healthcare outcome [2].

Maternal satisfaction refers to the satisfaction of mothers with delivery services and visit, duration of labour and mode of delivery [3]. It is also defined as women’s feeling about their caregivers [4]. Maternal satisfaction also includes expectation, perception of the aspect of labour, perceived control and having an active say in decision-making for the mother [5]. The important construct of birth satisfaction correlates with the childbearing women’s quality of care, personal attributes and stress experienced during labour [6].

To assess women’ satisfaction with labour and childbirth, various tools have been created. These include Women’s Views of Birth Postnatal Satisfaction Questionnaire (WOMBPNSQ) [7], Maternal Satisfaction [8] and Satisfaction with Antenatal Care, Satisfaction with Intrapartum Care and Satisfaction with Postnatal Care Questionnaire [9], which assess satisfaction with childbirth as a multidimensional construct [10]. The Satisfaction with Antenatal Care, Satisfaction with Intrapartum Care and Satisfaction with Postnatal Care Questionnaire assessed the impact of the new midwifery team system on antenatal, intrapartum and postnatal care satisfaction [9]. It was categorized into three domains, which are communication and relationship (35%), communication and the provision information (35%) and the woman’s assessment regarding the quality of care (30%). It consisted of 30-Likert style items, where eight of them focused on antenatal, ten focused on intrapartum and twelve on postnatal care.

The Maternal Satisfaction Questionnaire explored the effectiveness of the Maternity Group Practice and satisfaction with the model of care [8]. The items included two open-ended items followed by 8-Likert style items with three subscales such as continuity care, accessibility and personal and professional attributes of the midwife [11]. The WOMBPNSQ is a multidimensional and the dimensions were measured against the transformed dimension of general satisfaction. It is considered friendly questionnaires compared to others because of the familiar terms used, easily understood, a considerable number of items and suitable for self-administration. It can also be used to evaluate and contrast satisfaction with various care models or service configurations, as well as to assess changes over time [7].

Satisfaction evaluation offers critical performance information, thus leading to complete quality management [12]. Complete quality management includes professional knowledge, competence and application of suitable technology, perception of the type and level of care received by patients [13]. Most of the studies focused on antenatal satisfaction [14, 15], and one study looked at the delivery satisfaction following anesthesia [16]. To our knowledge, there was no questionnaire evaluating labour satisfaction, and this is the first postnatal satisfaction survey tested in the local community. The objective of this research was to evaluate the psychometric properties of the WOMBPNSQ version of Malay on postnatal satisfaction based on the Rasch scale model. It is founded on probabilistic and inferential item response theory. It focuses on the item response pattern that defines a person’s relationship with an item based on a shared latent trait [17].

## Materials and methods

### Population and sample

A cross-sectional study was carried out among postpartum women enrolled at hospital discharge in a referral hospital in Kota Bharu, Kelantan from July to September 2017. The study included women age 18 years and above, with a singleton pregnancy, underwent vaginal delivery and capable of speaking and understanding the Malay language was included. Those with abnormal lie pregnancy and medical illness were excluded. Non-probability sampling was applied. The sample size was based on a 99% confidence ½ logit with the best to poor sample size between 108 and 243 [18] and 195 postpartum women recruited for this study.

### Research tool

The WOMBPNSQ measures postpartum maternal satisfaction care. It contains 13 dimensions with 36 items, including a general satisfaction dimension (3 items). The remaining 12 dimensions are medical support (3 items), relationship support (3 items), social support (3 items), general practitioner care (2 items), health visitor care (2 items), contraceptive advice (3 items), baby feeding (4 items), mother’s health (3 items), continuity of care (2 items), duration of stay in hospital (3 items), home visit (3 items) and birth pain (2 items). Each item loaded highly onto one dimension. The original English version showed adequate overall reliability with Cronbach’s alpha of 0.84, and each dimension had acceptable or good internal reliability with Cronbach’s alpha ranging from 0.62 to 0.90 [7].

The items are scored on a Likert scale of 7 points, from ‘totally disagree’ to ‘totally agree’. Items were worded very positively or negatively to maximize the capacity of the respondent to convey dissatisfaction. The total score is derived by summing all item scores, and dimension scores are meant to facilitate easy comparisons between dimensions. The total score is produced by summing the scores of all items. The scores will be totaled by reversing the scores of negatively worded items and turning them to percentage scores, with the lowest possible score being zero and the greatest possible score being 100%. Higher scores indicate a higher level of satisfaction.

The WOMBPNSQ underwent a high-quality translation process based on the 10 recommended steps of the Translation and Cultural Adaptation-Principles of Good Practice. It begins with contacting the original author for approval to use the tool. A forward translation from English to Malay was carried out. The initial consensus was reached after the two forward translations were harmonised. The first consensus was followed by a backward translation. A linguist and a medical doctor assisted on each translation. The back-translation review compared the back-translated versions of the tool to the original in order to discover and address inconsistencies between the original and the reconciled version, which were subsequently changed to fix the issues.

The reverse translation of multiple language versions and the original tool were harmonized to highlight the discrepancies between the original and its derivative translation. Reviewing the instrument on six respondents served as cognitive debriefing, allowing us to examine alternative wording and ensure that the translation was comprehensible, understandable, and culturally relevant. Study of cognitive debriefing findings and finalization by contrasting the understanding the translation by laypeople with the original version was done to draw attention to and rectify disparities. Proofreading was done on the final translation review to find and amend any typographical, grammatical, or other mistakes. At the completion of the procedure, a final report detailing the progress of each translation was created [19]. We removed the home assessment dimension for the Malay version because it is not relevant locally, and the general satisfaction dimension was assessed independently of the other satisfaction dimensions [20]. As a result, 11 dimensions with 30 items were investigated (Supplementary file 1).

### Data collection

In obstetric wards, eligible postpartum women were identified. They were given a briefing and invited to take part in the research. Informed consent was taken once they agreed to participate. At 1-month postpartum, telephone interview of Malay version WOMBPNSQ on postnatal satisfaction was performed. Sociodemographic characteristics were obtained from the patients. Participation in this research does not pose a predictable or potential risk.

### Statistical analyses

Postnatal satisfaction was measured using the Rasch Polytomous model. The Rasch Polytomous model predicts how likely a person is to choose a specific response category or item. The Rasch Measurement Model transforms the raw data into equal interval units called logits [21] by applying log transformations and probabilistic equations. The relationship between the person’s measurement, the difficulty of the question, and the threshold of the score category determines the individual’s likely score. Winsteps version 3.72.3 [22] was used for Rasch data analysis.

Reliability of person and item shows to what degree the items are consistent (conform to fit) with the Rasch Model and the separation index of item and person. Reliability values > 0.8 are acceptable, whereas values 0.6 to 0.8 are less acceptable and values < 0.6 are not acceptable [21]. In cases of low reliability indices, they can be enhanced by giving special attention to the item or person misfits. Item and person separation index is for assessing questionnaire functioning. The person separation index indicates how well the items can separate or discriminate between individuals and how well the individual’s skill or trait can separate or discriminate between items [23]. The acceptable value of the separation indices > 2.0, resulting in a value of > 0.8 for the reliability of person and item [21, 24, 25]. By first contrast, a value of unexplained variance of less than 3% is excellent, 3-5% is very good, 5 to 10% is good, 10 to 15% is fair, and more than 15% is poor [26].

When an item fails to meet the goodness of fit criteria, such as point-measure correlation (PtMea Corr), outfit mean square (MnSq), or outfit z-score standardized (z-std) values, it is considered a misfit. A PtMea Corr value of 0.4 to 0.8 is considered acceptable. If the PtMea Corr value is < 0.3, this indicates that the items do not follow the criteria set [21]. The Likert scale’s acceptable range of fit is 0.5 to 1.5, implying that it is productive for measuring scale development. Values below 0.5 imply that the system is less productive but not degrading. Values between 1.5 and 2.0 indicate unproductive but not degrading, while values more than 2.0 distort or degrade the scale [27]. When the items are out of range, adjustments, or rephrasing must be made [25]. It is because of the matter that an item’s suitability can impact and influence the tool’s reliability and validity. The z-std represent a standardized sum of all the variations between observed and expected values summed up for all individuals [28], and the outfit z-std values of − 2 to + 2 are acceptable [27].

## Results

There were a total of 195 postpartum women participated in this study. All participants were married. The majority (95.4%) of the participants aged < 40 years old, had ≤ two children (62.6%), housewives (41.0%) and had tertiary education (48.2%).

### Person and item reliability

The summary data from the Women’s Views of Birth Postnatal Satisfaction Questionnaire for 195 persons and 30 items are shown in Table 1. The Person Raw Score of Cronbach Alpha (KR-20) was 0.50.

**Table 1:** Initial analysis of person and item summary statistics

	Person (*n* = 195)	Item (*n* = 30)
Cronbach’s alpha (α)	0.50	
Reliability index (μ)	0.45	0.99
Separation index	0.90	9.02
Mean	0.09	0.00
Max measure	0.70	0.54
Min measure	−0.31	− 0.67
Spread	1.01	1.21
Standard deviation	0.16	0.42
Outfit		
Mean Square	1.01	1.01
z-Standard	−0.20	0.20

The item reliability is 0.99, with a standard error of 0.08. Since the values are near to 1.0, the indices show that the items are very good. Person reliability is 0.45, which is somewhat higher than the expected model of 0.54 with a standard error of 0.01.

### Separation index

The person’s ability to discriminate the 30 items into nine strata or levels of agreement is excellent, with an Item separation index of 9.02 (Table 1). However, the items cannot discriminate the high from low performers as indicated by the person separation index of 0.90 (Table 1).

### Unidimensionality

Unidimensionality is a critical component that determines construct validity. The residuals can be described using Principal Component Analysis (PCA) of the residuals, as illustrated in Table 2. The raw variance explained by measures in Residuals’ Rasch PCA was 42.6%, which was similar to the model’s predicted variance. (42.6%) with the 7.7% noise in the item. The unexplained variance between 10 and 15% in the first contrast is a fair indicator of unidimensionality.

**Table 2:** Standardized residual variance using Principal Component Analysis

Standardized residual variance (in Eigenvalue units)	Eigen	Empirical (%)
Total raw variance in observations	52.2	100.0
Raw variance explained by measures	22.2	42.6
Raw variance explained by persons	1.8	3.4
Raw variance explained by items	20.5	39.2
Raw unexplained variance (total)	30.0	57.4
Unexplained variance in 1st contrast	4.0	7.7

### Item dependency

Local dependency tests for the greatest standardised residual correlation yield an excellent result, with none of the items exceeding the 0.70 threshold, showing item independence (Table 3). For example, for the vast majority of respondents, item CA_Q30 ‘*My carers discussed the full range of contraception options following the birth of my baby’* has material effect on item FB_Q31 *‘I was given lots of help on how to feed my baby’*.

**Table 3:** Largest standardized residual correlations for items

Correlation	Item	Item
0.49	CA_Q30	FB_Q31
0.49	CA_Q4	PNV_Q7
0.45	FB_Q36	MH_Q16
0.42	IPS_Q28	FB_Q5
0.36	CA_Q4	HS_Q6
0.36	CON_Q25	GPC_Q26
−0.44	HVC_Q24	PAB_Q23
−0.39	CA_Q4	FB_Q5
−0.37	CA_Q17	PNV_Q20
−0.37	GPC_Q26	AB_Q23

The response pattern between item CA_Q30 and item FB_Q31 is 49% similar. The person answered 7 for item CA_Q30 has answered 7 for item FB_Q31 on a very high level. Similarly, the person answered 2 for item CA_Q30, answered 3 for item FB_Q31, at the lowest level. This showed unidimensionality with strong internal consistency, and it’s a reliable tool for determining what it meant.

### Goodness of fit test

The overall outfit MnSq and outfit z-std were 1.01 and 0.20, respectively, according to the Item fit statistics analysis (Table 1), which is within the expected ranges of 1.00 and 0.00. It indicates the tool’s goodness of fit, and that it measures what is to be measured. The statistics on Item Misfit were subjected to a second investigation. The parameters for the statistics concerning 30 items reported measures between 0.54 *logit* and − 0.67 *logit.* The outfit MnSq was 1.43 to 0.79*,* the outfit z-std was 5.9 to − 1.80*,* and the PtMea Corr was 0.53 to − 0.04*.*

Based on item misfit order, none of the item fulfil all the three criteria for outfit MnSq, outfit z and PtMea Corr; and are considered fit. The MnSq indicates that all of the items can be used to build a measuring scale. The PtMea Corr indicates that only five items correlate with the construct.

### Person misfit

The MnSq and z-std overall outfits were determined to be 1.01 and − 0.20, respectively, in the Person fit statistics analysis, which is extremely close to the 1.00 and 0.00 expectations (Table 1). It demonstrates that the 30 items target the correct respondents, have a small amount of distortion in the measurement of latent traits, and that the data obtained have a good level of predictability of the responses to the items. To verify that the 195 people were fit, a second analysis of the person misfit statistics was conducted. The parameters for the statistics concerning 195 persons reported measures between 0.67 and-0.20 *logit.* The outfit MnSq was 4.39 to 0.14*,* the outfit z-std was 5.40 to − 4.60*,* and the PtMea Corr was 0.90 to − 0.39*.*

Based on item IPS Q15, five people (entry numbers 191, 186, 164, 61, and 134) are identified to have the most mismatched response strings. These five people were excluded because they provided noise to the measuring process and were of no psychometric importance. The WOMBPNSQ was fitted twice to test the effect of misfitting: once with all items and people, and then again with five people removed from the model. Based on our findings, there is no difference in the parameter estimates, and therefore, the five persons are retained in the model.

### Wright item-person map

**Fig. 1:**
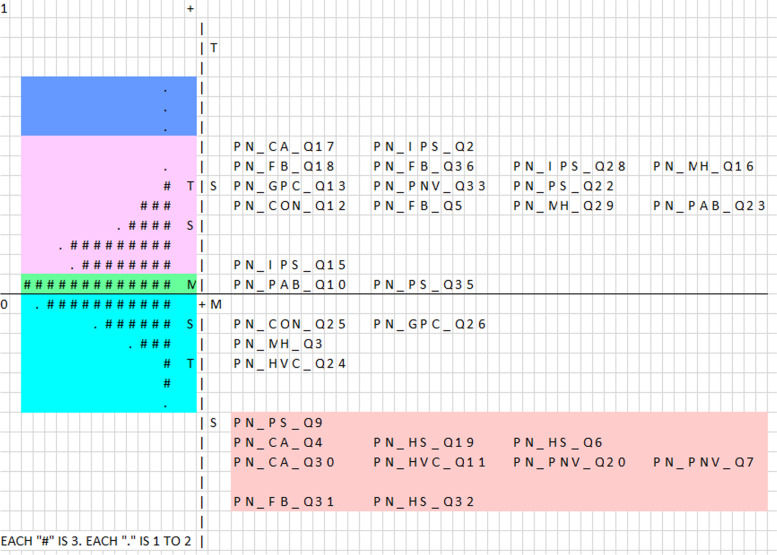
Wright item-person map of Women’s Views of Birth Postnatal Satisfaction Questionnaire

On the logit scale, Fig. 1 shows the number of respondent abilities and the difficulty of the item. All of the items are spread, indicating the respondents’ ability to be diverse. The item mean is set to 0.00 logit by default, ensuring that each group of people has a 50:50 chance of reacting to the item that matches their ability, while the Person mean is set at 0.09 logit. Because the mean of both values is roughly identical, the items in this sample are well-targeted.

With a 1.21 logit spread, the item difficulty ranges from + 0.54 logit to − 0.67 logit. Furthermore, with a 1.01 logit spread, the Person ability estimations range from + 0.70 logit to − 0.31 logit. When compared to the spread of the item, the person’s dispersion has a considerably smaller spread. The limited distribution of people indicates a shared latent trait of postpartum women and a common experience of satisfaction. Respondents who were dissatisfied were found at the top of the map, while those who were satisfied were found at the bottom. All women are easily satisfied with 10 of the items (Table 4). The least satisfied item is CA_Q17 (0.54 logit) *‘I was given little advice on contraception following the birth of my baby’* at 0.54 logit. There are only four persons who are less satisfied (above 0.54 *logit*) (Supplementary file 2).

**Table 4:** Easily endorsed items for postnatal satisfaction

Logit	Item	Description
− 0.45	PS_Q9	My carers were never insensitive nor lacked understanding
− 0.46	HS_Q19	I could have had just a very little more help from my birth partner/husband
−0.46	CA_Q4	My carers explored adequately with me my contraceptive needs
−0.45	HS_Q6	My partner met all my needs after the birth
−0.51	PNV_Q7	The visits I received in my home were always convenient
−0.54	PNV_Q20	My postnatal check-ups were always at a very convenient time
−0.56	CA_Q30	My carers discussed the full range of contraception options following the birth of my baby
−0.56	HVC_Q11	The health visitors were really good at helping me to feed my baby
−0.66	FB_Q31	I was given lots of help on how to feed my baby
−0.67	HS_Q32	My partner/husband was the best possible help to me after the baby was born

## Discussion

Fit is a quality control principle that reflects how well the data fits the model accurately and predictably. This stage is necessary for assessing the fit of each item’s measures to allow much farther analysis. The Malay version WOMBPNSQ on postnatal satisfaction has a low value of Cronbach Alpha (0.50), indicating poor internal consistency reliability of items in the scale when measuring a construct or single latent trait. However, the item reliability was high, which showed that the items’ high reliability suggested that there would be a 99% likelihood of reproducibility of the items if the tool were to be given to another population of the same size [21]. Low reliability values for persons indicate a limited range of person measures or a small number of items. Whereas, low reliability for items suggests a limited range of item measures or a small sample. This sample shows low reliability of persons but high reliability of items, indicating a narrow range of measures of persons but with an adequate sample [29]. One possible reason may be because of the homogenous population. It consists of postpartum women who lived in a setting where the demographic in relation to education, income, were approximately similar. Therefore, this study suggests that people with more extreme abilities (high and low) be included to improve targeting for the test.

The outstanding item separation index (9.02) revealed that the 30 items in the measurement of birth postnatal satisfaction have a good spread. It also reveals the instrument’s goodness-of-fit and thus its validity in measuring what is supposed to be measured. The items can also be used to categorize people into one group. Because the items are divided by levels of varied difficulty, the higher the value of the items separation index, the better the measurement tool. When the reliability of items increases, the separation index will increase, and misfit items are detected and removed from the analysis. These indices show that the difference or separation between persons can measure persons’ ability [21, 30].

One of the key assumptions for the data to match the Rasch model is unidimensionality. Construct requires ≥ five items before the factor or construct is treated as a different dimension [25]. The lack of any meaningful pattern in the residuals indicates unidimensionality [31]. Unidimensionality refers to features of test items measuring a single ability. Unidimensionality is a critical component that determines construct validity. It can be described using the PCA. To satisfy unidimensionality, items in the instruments must measure the same composite of abilities, i.e. postnatal satisfaction.

Variance components are rescaled so that the total unexplained variance has its expected summed eigenvalue. The eigenvalue units are rescaled to match the number of items so that the values are equivalent to strength in item units. The measures explained the amount of variance in the data. It fits the model and accorded with the Rasch definition of unidimensionality [29]. The raw variance explained by measures was 42.6%, which was equal to the model’s expected variance, according to Residuals’ Rasch PCA. It’s low, yet it meets the minimum unidimensionality threshold of 40%, indicating a strong measurement dimension [32]. The unexplained variance between 10 and 15% in the first contrast is a fair indicator of unidimensionality [26]. As a result, when measuring birth-level satisfaction, the existing 30 items can be viewed as one-dimensional. The greatest standardized residual correlation identifies item dependency or multicollinearity. Locally dependent item pairs with a high correlation coefficient (> 0.7) can be considered redundant.

Rasch goodness of fit based on item polarity and misfit indicates how well an item fits the model. It helps researchers to detect misfit items to refine a test tool further. Item polarity is a point-measure correlation whereby properly functioning items should exhibit a positive correlation coefficient. In interpreting the measured constructs by the positive PtMea Corr value, all items must move in a similar direction. If PtMea Corr’s value is high, it indicates that the items can differentiate between respondents’ ability [21]. The Items fit, based on the outfit MnSq and z-std were examined to test each item’s goodness of fit to the Rasch model. The data’s fitness was tested to ensure that the items are in linear interval scale measures on the *logit* continuum scale. The outfit and infit MnSq statistics was used to investigate whether all items contribute equally to their domain. They are used to assess the content validity of the survey items [33]. The MnSq infit is a weighted index that notes the difference between the observed and expected response for those items that have difficulty close to the ability level of the person, while outfit MnSq is an unweighted index that includes differences for all items regardless of how far the difficulty of the item is from the ability of the person [28].

By the pattern of the responses, the possible causes might be guessing or carelessness. These five people were excluded because they provided noise to the measuring process and were of no psychometric importance [24]. The WOMBPNSQ was fitted twice to test the effect of misfitting: once with all items and people, and then again with five people removed. Based on our findings, there is no difference in the parameter estimates, and therefore, the five persons are retained in the model.

Along the logit scale, the Wright item-person map illustrates locations of item difficulty and person distribution. It aids in locating the area where the majority of the items are placed, particularly in determining whether this is parallel to the respondents’ dispersion. When a set of items in a domain is capable of covering the whole population-scale score range, it is said to be optimally targeted. The item difficulty mean should be near to the mean scale ratings of the respondents, and a larger disparity in the means correlates to poorer targeting [34].

We note several limitations to our study. First, because the women were interviewed in Malay, the study’s generalizability to other communities may be limited. Next, they were contacted through phone calls which may limit the responses to the items. Rasch’s first attempt to analyze multiple-choice items, which measure women’s capacity to estimate postpartum care satisfaction, is in this study. All of the items fit the model well and can be used in the environments where they were evaluated.

## Conclusion

The WOMBPNSQ has a low person reliability index and a high item reliability index when conducted among mothers at 1-month postpartum. Person fit statistics are very near to the expectation of 1.00 and 0.00. Compared to item spread, it has a narrow spread of person. It shows that the 30 items target the right respondents, have similar latent features of postpartum women and a shared sense of satisfaction. For future improvement, more difficult items endorsing satisfaction should be created, and the common items in which satisfaction is expected should be reduced.

## Supplementary Information

**Additional file 1.**

**Additional file 2.**

## Data Availability

All data are available within the manuscript.

## Abbreviations

MnSq: Outfit mean square
LID: Local item dependency
PCA: Principal Component Analysis
PtMea Corr: Point-measure correlation
SE: Standard error
WOMBPNSQ: Women’s Views of Birth Postnatal Satisfaction Questionnaire
z-std: Outfit z-score standardized
