# Supplementary material for: Psychometric properties of the Malay version Women’s Views of Birth Postnatal Satisfaction Questionnaire using the Rasch measurement model: a cross-sectional study
Source: BMC Pregnancy Childbirth. 2021 Oct 22;21:711. doi: 10.1186/s12884-021-04184-8 (PMC8532326; doi:10.1186/s12884-021-04184-8)
Supplement: Supplementary file 1 — Additional file 1. [file 12884_2021_4184_MOESM1_ESM.pdf]

Supplementary file 1. Women’s Views of Birth Postnatal Satisfaction Questionnaire in English and Malay languages

WOMEN'S VIEWS OF BIRTH POSTNATAL SATISFACTION QUESTIONNAIRE
SOAL-SELIDIK PANDANGAN WANITA TERHADAP KEPUASAN SELEPAS BERSALIN

Instruction:
Arahan:
For each of the following statement, please indicate how it relates to you based on the following scale:
Untuk setiap penyataan, sila tandakan bagaimana ia menggambarkan keadaan anda:

		Strongly agree	*Agree*	*Slightly agree*	*Undecided*	*Slightly disagree*	*Disagree*	*Strongly disagree*
Item	*Description* Penerangan	Sangat setuju	Setuju	Kurang bersetuju	Tidak pasti	Kurang tidak bersetuju	Tidak bersetuju	Sangat tidak bersetuju
Item 2 (IPS_Q2)	*I could have done with more time for my body to adjust after the birth before going home* Saya sepatutnya diberi masa yang lebih untuk menyesuaikan diri selepas kelahiran sebelum dibenarkan pulang ke rumah							
Item 25 (MH_Q3)	*Everyone concentrated just on my physical health after the birth and not on how I was feeling* Semua orang mengambil berat tentang kesihatan fizikal saya tanpa mengambil kira perasaan yang saya alami							
Item 4 (CA_Q4)	*My carers explored adequately with me my contraceptive needs* Staf kesihatan memberi penerangan yang secukupnya tentang keperluan pencegahan kehamilan yang saya perlukan atau yang bersesuaian dengan saya							
Item 7 (FB_Q5)	*I would have liked more time to discuss feeding problems during carers' visits* Saya memerlukan lebih masa untuk membincangkan masalah pemakanan bayi semasa lawatan staf kesihatan							
Item 11 (HS_Q6)	*My partner met all my needs after the birth* Suami saya memenuhi semua keperluan saya selepas bersalin							

Item 26 (PNV_Q7)	*The visits I received in my home were always convenient*<br>Lawatan di rumah yang saya terima sentiasa bersesuaian							
Item 14 (PS_Q9)	*My carers were never insensitive nor lacked understanding*<br>Staf kesihatan saya tidak pernah tidak peka atau tidak memahami							
Item 29 (PAB_Q10)	*I didn't need a lot of pain relief after the birth*<br>Saya tidak memerlukan ubat penahan kesakitan yang banyak selepas bersalin							
Item 17 (HVC_Q11)	*The health visitors were really good at helping me to feed my baby*<br>Staf kesihatan yang membuat lawatan ke rumah membantu saya dalam penyusuan bayi							
Item 20 (CON_Q12)	*I was usually visited at home by different carers*<br>Saya sentiasa dilawati oleh staf kesihatan yang berlainan							
Item 22 (GPC_Q13)	*My GP had no role in my postnatal care*<br>Doktor di klinik tidak memainkan peranan dalam rawatan selepas kelahiran							
Item 2 (IPS_Q15)	*It would have been so much better if I had had a longer hospital stay after the birth*<br>Adalah lebih baik jika saya dibenarkan berada lebih lama di hospital selepas bersalin							
Item 23 (MH_Q16)	*A little more time being spent on my health would have been welcome*<br>Saya berharap untuk mendapat penjagaan kesihatan yang lebih ke atas diri saya							
Item 5 (CA_Q17)	*I was given little advice on contraception following the birth of my baby*<br>Saya diberikan sedikit sahaja penerangan berkenaan pencegahan kehamilan selepas kelahiran bayi							
Item 8 (FB_Q18)	*Sometimes I was given conflicting advice from health visitors and/or other carers*							

	Kadangkala saya mendapat nasihat yang berbeza daripada staf-staf kesihatan							
Item 12 (HS_Q19)	*I could have had just a very little more help from my birth partner/husband*<br>Saya memerlukan lebih sedikit sahaja lagi bantuan daripada suami saya							
Item 27 (PNV_Q20)	*My postnatal check-ups were always at a very convenient time*<br>Pemeriksaan selepas kelahiran saya sentiasa dijalankan pada masa yang sesuai							
Item 15 (PS_Q22)	*I sometimes had problems understanding what my carers were saying to me*<br>Kadang-kadang saya tidak faham apa yang staf kesihatan ingin sampaikan kepada saya							
Item 30 (PAB_Q23)	*I was in a fair bit of pain in the first few days/weeks after the birth*<br>Saya berada dalam keadaan yang agak sakit beberapa hari / minggu selepas bersalin							
Item 18 (HVC_Q24)	*The caring approach of the health visitor really helped me and my new baby*<br>Pendekatan secara penyayang yang ditunjukkan oleh staf kesihatan yang melawat sangat membantu saya dan bayi saya							
Item 19 (CON_Q25)	*I saw the same carer at postnatal visits rather than different ones each time*<br>Saya dilihat oleh staf kesihatan yang sama semasa rawatan selepas kelahiran, bukannya staf yang berlainan pada setiap lawatan							
Item 21 (GPC_Q26)	*My GP was really helpful in the weeks after the birth*<br>Doktor di klinik sangat membantu saya dalam beberapa minggu selepas kelahiran							

Item 3 (IPS_Q28)	*I needed more time in hospital to get used to caring for my new baby*<br>Saya memerlukan masa yang lebih lama untuk menyesuaikan diri dan mahir dalam penjagaan bayi								
Item 24 (MH_Q29)	*I would have liked more chance to talk to my carers for medical advice about care of myself*<br>Saya berharap mendapat lebih peluang untuk berbincang dengan staf kesihatan tentang penjagaan diri saya								
Item 6 (CA_Q30)	*My carers discussed the full range of contraception options following the birth of my baby*<br>Staf kesihatan membincangkan secara terperinci tentang kaedah pencegahan kehamilan yang sesuai selepas melahirkan bayi								
Item 9 (FB_Q31)	*I was given lots of help on how to feed my baby*<br>Saya diberikan tunjuk ajar yang baik bagaimana untuk menyusukan bayi saya								
Item 13 (HS_Q32)	*My partner/husband was the best possible help to me after the baby was born*<br>Suami saya merupakan orang yang terbaik untuk memberi bantuan selepas bayi dilahirkan								
Item 28 (PNV_Q33)	*The visiting times of health visitors were sometimes inconvenient*<br>Masa staf kesihatan melakukan lawatan kadang-kadang tidak sesuai								
Item 16 (PS_Q35)	*The carers who treated me should sometimes have given me just a little more respect*<br>Staf kesihatan yang merawat saya sepatutnya lebih menunjukkan rasa hormat kepada saya								
Item 10 (FB_Q36)	*I would have liked more advice on feeding my baby*<br>Saya suka sekiranya diberikan penerangan yang lanjut berkaitan penyusuan bayi saya								
