# Supplementary material for: Psychometric properties of the Malay version Women’s Views of Birth Postnatal Satisfaction Questionnaire using the Rasch measurement model: a cross-sectional study
Source: BMC Pregnancy Childbirth. 2021 Oct 22;21:711. doi: 10.1186/s12884-021-04184-8 (PMC8532326; doi:10.1186/s12884-021-04184-8)
Supplement: Supplementary file 2 — Additional file 2. [file 12884_2021_4184_MOESM2_ESM.pdf]

Supplementary file 2. Fit statistics for items

TABLE 10.1 WOMBPNSQ ZOU512WS.TXT Nov 26 14:57 2019
INPUT: 195 Person 30 Item REPORTED: 195 Person 30 Item 7 CATS WINSTEPS 3.72.3

Person: REAL SEP.: .90 REL.: .45 ... Item: REAL SEP.: 9.02 REL.: .99

Item STATISTICS: MISFIT ORDER

ENTRY	TOTAL	TOTAL		MODEL	INFIT		OUTFIT		PT-MEASURE		EXACT MATCH		
NUMBER	SCORE	COUNT	MEASURE	S.E.	MNSQ	ZSTD	MNSQ	ZSTD	CORR.	EXP.	OBS%	EXP%	Item
2	739	195	.15	.04	1.36	5.3	1.43	5.9	A .00	.30	9.2	9.5	PN_IPS_Q15
30	623	195	.31	.04	1.16	2.1	1.30	3.4	B-.04	.30	19.0	12.9	PN_PAB_Q23
23	513	195	.49	.04	1.22	2.0	1.11	.9	C .53	.28	22.1	26.6	PN_MH_Q16
25	962	195	-.15	.04	1.17	2.0	1.22	2.2	D .10	.26	18.5	16.7	PN_MH_Q3
29	821	195	.04	.04	1.15	2.4	1.21	2.9	E .17	.29	6.2	9.1	PN_PAB_Q10
11	1120	195	-.46	.05	1.11	.8	1.11	.8	F .22	.20	31.3	37.5	PN_HS_Q6
12	1114	195	-.45	.05	.92	-.6	1.11	.7	G .03	.21	39.5	36.7	PN_HS_Q19
8	533	195	.46	.04	1.07	.7	1.10	.9	H .16	.28	26.7	22.8	PN_FB_Q18
20	596	195	.35	.04	1.08	.9	1.09	1.0	I .25	.29	11.3	14.7	PN_CON_Q12
14	1102	195	-.42	.05	.95	-.3	1.05	.4	J .20	.21	43.6	34.0	PN_PS_Q9
22	550	195	.43	.04	1.02	.3	1.04	.5	K .27	.29	28.2	20.2	PN_GPC_Q13
19	919	195	-.09	.04	.99	-.1	1.04	.5	L .20	.27	17.9	13.0	PN_CON_Q25
6	1154	195	-.56	.06	.94	-.3	1.03	.2	M .23	.18	38.5	41.0	PN_CA_Q30
28	570	195	.39	.04	1.02	.3	.97	-.3	N .25	.29	22.6	17.2	PN_PNV_Q33
4	1119	195	-.46	.05	.95	-.3	1.02	.2	O .32	.20	47.2	37.5	PN_CA_Q4
24	608	195	.33	.04	1.01	.1	.98	-.2	o .46	.29	13.3	13.8	PN_MH_Q29
7	625	195	.31	.04	.98	-.3	.98	-.2	n .31	.30	12.8	12.4	PN_FB_Q5
27	1149	195	-.54	.06	.98	-.1	.96	-.2	m .28	.19	40.0	40.8	PN_PNV_Q20
13	1185	195	-.67	.06	.75	-1.4	.98	-.1	l .04	.16	54.9	41.7	PN_HS_Q32
10	513	195	.49	.04	.96	-.3	.93	-.6	k .43	.28	34.4	26.6	PN_FB_Q36
17	1155	195	-.56	.06	.94	-.3	.86	-.8	j .37	.18	41.5	41.0	PN_HVC_Q11
15	562	195	.41	.04	.92	-.9	.94	-.6	i .18	.29	28.2	17.9	PN_PS_Q22
3	514	195	.49	.04	.93	-.6	.92	-.7	h .38	.28	28.2	26.6	PN_IPS_Q28
1	506	195	.51	.04	.91	-.8	.92	-.7	g .44	.28	37.4	27.5	PN_IPS_Q2
26	1137	195	-.51	.05	.84	-1.1	.90	-.6	f .19	.19	45.6	39.7	PN_PNV_Q7
18	984	195	-.19	.04	.85	-1.7	.88	-1.3	e .27	.26	19.0	17.9	PN_HVC_Q24
16	819	195	.05	.04	.86	-2.3	.86	-2.3	d .37	.29	14.4	9.1	PN_PS_Q35
21	880	195	-.04	.04	.85	-2.3	.84	-2.3	c .44	.28	10.8	10.7	PN_GPC_Q26
9	1176	194	-.66	.06	.81	-1.0	.85	-.8	b .27	.17	46.4	41.7	PN_FB_Q31
5	490	195	.54	.05	.80	-1.8	.79	-1.8	a .30	.27	39.0	30.3	PN_CA_Q17
MEAN	824.6	195.0	.00	.05	.98	.0	1.01	.2			28.2	24.9	
S.D.	261.4	.2	.42	.01	.13	1.6	.14	1.6			13.3	11.5	
